# The Heat Shock Protein 70 Plays a Protective Role in Sepsis by Maintenance of the Endothelial Permeability

**DOI:** 10.1155/2020/2194090

**Published:** 2020-09-05

**Authors:** Xiaoyan Yuan, Yajing Chen, Guo Chen, Guorong Liu, Min Hang, Pei Wang, Yajuan Luo, Dongfeng Guo, Lei Xu

**Affiliations:** ^1^Department of Emergency Medicine, Gongli Hospital of Shanghai Pudong New Area, China; ^2^Ningxia Medical University, China

## Abstract

Sepsis is a severe system inflammatory response syndrome in response to infection. The vascular endothelium cells play a key role in sepsis-induced organ dysfunction. The heat shock protein 70 (HSP70) has been reported to play an anti-inflammatory role and protect from sepsis. The present study is aimed at finding the function of HSP70 against sepsis in vascular endothelium cells. Lipopolysaccharide (LPS) and HSP70 agonist and inhibitor were used to treat HUVEC. Cell permeability was measured by transepithelial electrical resistance (TEER) assay and FITC-Dextrans. Cell junction protein levels were measured by western blot. Mice were subjected to cecal ligation and puncture (CLP) to establish a sepsis model and were observed for survival. After LPS incubation, HSP70 expression was decreased in HUVEC. LPS induced the inhibition of cell viability and the increases of IL-1*β*, IL-6, and TNF-*α*. Furthermore, cell permeability was increased and cell junction proteins (E-cadherin, occludin, and ZO-1) were downregulated after treatment with LPS. However, HSP70 could reverse these effects induced by LPS in HUVEC. In addition, LPS-induced elevated phosphorylation of p38 can be blocked by HSP70. On the other hand, we found that inhibition of HSP70 had similar effects as LPS and these effects could be alleviated by the inhibitor of p38. Subsequently, HSP70 was also found to increase survival of sepsis mice *in vivo*. In conclusion, HSP70 plays a protective role in sepsis by maintenance of the endothelial permeability via regulating p38 signaling.

## 1. Introduction

Sepsis is defined as a system inflammatory response syndrome (SIRS) to infection [1]. The invasion of pathogenic bacteria or opportunistic pathogens and their continuous reproduction in the blood circulation cause severe systemic infection, which may progress to septic shock, multiple organ failure, and death [2]. Recently, dysfunction of the endothelial system has been found to be detrimental to the recovery of septic patients and it can also lead to the dysfunction of multiple organs [3, 4]. The increase of vascular endothelial permeability is the main mechanism of capillary leak syndrome, which can cause high mortality in early stages of sepsis [5]. Although the specific mechanism is not clearly established yet, the improvement of vascular endothelial cell function has a certain inhibitory effect on the body injury caused by sepsis [6, 7]. Therefore, it has great clinical significance to seek new targets related to the regulation of vascular endothelial cells for the prevention and treatment of sepsis.

The vascular endothelium participates in the following: vascular barrier, vascular integrity, inflammation regulation, blood coagulation, and maintenance of homeostasis [8]. Usually, the vascular endothelium forms a continuous, semipermeable barrier which regulates the transition of cellular molecules [9]. In sepsis, the vascular endothelium is activated by lipopolysaccharides (LPS) and/or inflammatory mediators including interleukin-6 (IL-6), tumor necrosis factor-*α* (TNF-*α*), and interleukin-1 (IL-1) [10, 11]. The increase of inflammation caused by sepsis contributes to the injury of vascular endothelial cells by decreasing vascular density and increasing vascular permeability, resulting in a shift of circulating elements and tissue edema even contributing to organ failure and mortality [12, 13]. Thus, regulating vascular permeability plays a key role in the treatment of sepsis.

The heat shock proteins (HSPs) are usually termed as stress proteins and have been reported to perform multiple protective functions [14]. HSP70, one of the well-studied HSPs, has been reported to facilitate protein folding and prevent the secretion of inflammatory mediators. HSP70 is also found to play an antiapoptotic and anti-inflammatory role in animal models for stroke [15, 16]. Additionally, pretreatment with HSP70 protects sepsis-induced acute lung injury and increases animal survival [17]. Moreover, HSP70 effectively downregulates the expressions of proinflammatory genes by interrupting the phosphorylation of transcription factors including p38 [18]. HSP70 is also reported to bind to and inhibit NF-*κ*B, which is a critical factor in the pathophysiology of sepsis [19]. However, the underlying mechanism of HSP70 in sepsis remains unclear. The current study was aimed at exploring the function of HSP70 in sepsis.

## 2. Materials and Methods

### 2.1. Cell Culture and Treatment

The HUVEC line was purchased from the American Type Culture Collection (ATCC; Manassas, VA, USA) and cultured in Dulbecco's Modified Eagle's Medium (DMEM; Gibco, Carlsbad, CA, USA) containing 10% fetal bovine serum (Gibco) and 1% penicillin/streptomycin (Invitrogen, Carlsbad, CA, USA) at 37°C, in 5% CO_2_.

Cells were treated with 0, 200, 500, 1000, and 2000 ng/ml LPS (Sigma, St. Louis, MO, USA) for 24 h or treated with 500 ng/ml LPS for 0, 6, 12, and 24 h; then, the concentration of HSP70 was measured. Further, cells were treated with 0, 6.25, 12.5, and 25 *μ*M TRC051384 (Tocris, Minneapolis, MN, USA), an agonist of HSP70, for 4 h; then, cells were treated with 500 ng/ml LPS for 24 h. Treated cells were used for other experiments. For cotreatment with Apoptozole (Tocris, Minneapolis, MN, USA), an inhibitor of HSP70, and SB203580 (Selleck, Radnor, PA, USA), an inhibitor of p38, cells were treated with 10 *μ*M SB203580 and 10 *μ*M Apoptozole for 20 h.

### 2.2. ELISA

The productions of HSP70, IL-1*β*, IL-6, and TNF-*α* were detected by ELISA kits (CUSABIO, Houston, TX, USA). The supernatant of treated cells (in triplicate) was collected and used for measuring the concentrations of these cytokines according to the manufacturer's protocol.

### 2.3. Real-Time PCR

Treated cells were harvested using the TRIzol reagent kit (Invitrogen, Carlsbad, CA, USA) to obtain total RNA, and then the cDNA synthesis kit (Promega, Madison, WI, USA) was used to reverse transcribe RNA to cDNA according to the manufacturer's instructions. The relative expression level of HSP70 was analyzed using SYBR Green qPCR Master Mixes (Thermo Fisher Scientific Inc., Grand Island, NY, USA) and primers (primer F 5′ AGTGGAGATAGTTGGTGGTG 3′; primer R 5′ TTGAAAGCAGGCGATAAG 3′) on an ABI 7300 system (Applied Biosystems, Foster City, CA, USA). GAPDH was used as an internal control using 2^-*ΔΔ*CT^, and each sample was performed in three duplications.

### 2.4. Western Blot

HUVEC were lysed on ice using the RIPA lysis buffer (Solarbio, Beijing, China), and cell lysates were centrifuged at 12,000 rpm at 4°C for 5 min. Then, the supernatant was collected for measuring the protein concentration using the bicinchoninic acid (BCA) protein assay kit (Thermo Fisher Scientific Inc., Grand Island, NY, USA) and then detecting protein expression. Proteins (25 *μ*g) were subjected to 10% SDS-PAGE gel then transferred onto a PVDF membrane (Millipore Corp., Bedford, MA, USA). After blocking in 5% nonfat milk for 2 h, the membranes were incubated with primary antibodies at 4°C overnight and then incubated with horseradish peroxidase- (HRP-) conjugated secondary antibody for 2 h. Finally, protein bands were visualized using the enhanced chemiluminescence (ECL) kit (Millipore, Burlington, MA, USA). ImageJ software was used to analyze the gray value of each band. Primary antibodies used were as follows: HSP70 (Ab5439, 1 : 800), occludin (Ab168986, 1 : 1000), and ZO-1 (Ab96587, 1 : 800) were purchased from Abcam; E-cadherin (#14472, 1 : 800), p-38 (#9212, 1 : 800), p-p38 (#9211, 1 : 800), and GAPDH (#5174, 1 : 2500) were purchased from CST (Cell Signal Technology).

### 2.5. Cell Viability

Cell viability was measured by CCK-8 assay (Beyotime, Shanghai, China). HUVEC were seeded into a 96-well plate in triplicate and cultured with different treatments. At each time point, their viabilities were measured. The optical density was measured at 450 nm by using an auto-microplate reader (Bio-Rad Laboratories, Inc., Hercules, CA, USA).

### 2.6. Transepithelial Electrical Resistance (TEER) Assay

TEER of HUVEC was measured using MERSST ×01 Electrode (EMD Millipore Corporation, Bedford, MA, USA). Treated cells were planted on 0.4 mm transwell filters coated with fibronectin at density of 1 × 10^6^ cells/ml. After full confluence, MERSST ×01 Electrode was used to detect TEER values of each group. The TEER value was shown as the common unit (V cm^2^), and a cell-free filter was used as the blank control [20]. Each sample was performed in triplicate.

### 2.7. Cell Permeability Measurements

The permeability of monolayers was performed as described previously [21]. Treated cells were planted on 0.4 mm transwell filters at density of 1 × 10^5^ cells/ml. After full confluence, the cell culture medium was replaced with DMEM (serum-free and phenol red-free) for 4 h. Then, 1 mg/ml FITC-Dextrans (Sigma, St. Louis, MO, USA) were added to the upper chamber and cultured for 1 h. Subsequently, 200 *μ*l samples were collected from the lower chamber and were measured on a fluorescence plate reader (excitation 490 nm, emission 520 nm). The concentration of permeable FITC-Dextrans was calculated according to standard curves, and each sample was performed in three duplications.

### 2.8. Animal Model

16-month-old male C57BL/6 mice were obtained from Shanghai SLAC Laboratory Animal Co., Ltd. (Shanghai, China), and the animal experiment was performed at the Experimental Animal Center of the Second Military Medical University. All the mice were housed under a 12 h light-dark cycle, with ad libitum access to food and water. All experimental procedures involving animals were approved by Gongli Hospital of Shanghai Pudong New Area (Shanghai, China) in accordance with the National Institutes of Health Guidelines.

All the mice were randomly divided into 4 groups (20 mice each group): control, cecal ligation and puncture (CLP), CLP+10 mg/kg Apoptozole, and CLP+10 mg/kg TRC051384. A sepsis model was established by the CLP procedure [22]. First, mice were anesthetized using isoflurane inhalation (Sigma, St. Louis, MO, USA). After disinfecting the abdomen, a 1 cm longitudinal skin midline incision was made in the lower abdomen to expose the cecum. Ligate the cecum at the desired position for midgrade sepsis. Then, the cecum was punctured thoroughly with a 22-gauge needle. To ensure patency, a droplet of feces could be squeezed out of the hole. Subsequently, the cecum was put back to the abdomen, which was closed by two-layer suturing. For the control, the cecum was exposed but not ligated or punctured and then returned into the original place. CLP model mice were observed every 8 h for 7 days for survival analysis, and surviving mice were euthanized at the 7^th^ day.

### 2.9. Statistical Analysis

Data were presented as mean ± SEM. Multiple comparisons between different groups were analyzed using one-way ANOVA followed by the least significant difference post hoc test. The survival rate in different groups was analyzed by Kaplan-Meier survival curves and log-rank statistics. GraphPad Prism 5 software was used for the statistical analysis, and *P* < 0.05 was considered statistically significant.

## 3. Results

### 3.1. HSP70 Was Downregulated by LPS Stimulation in HUVEC

**Figure 1:**
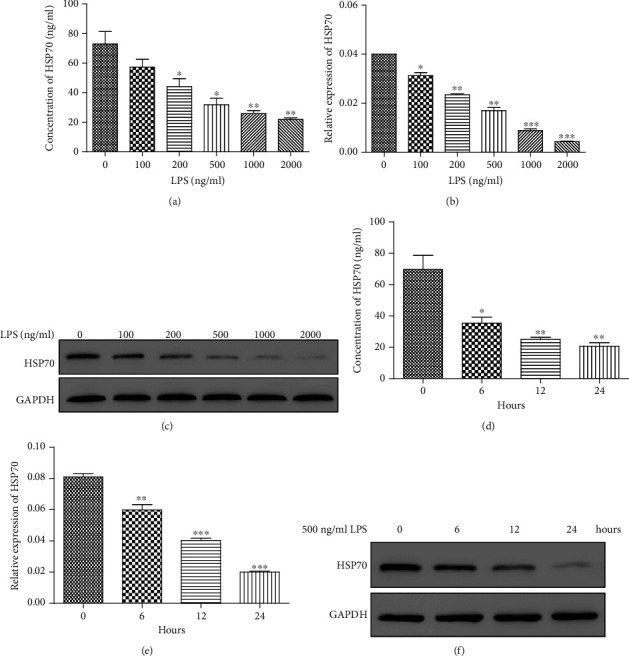
HSP70 level was detected after treatment with LPS. (a) ELISA was used to detect the secretory HSP70 after treatment with different concentrations of LPS (0, 200, 500, 1000, and 2000 ng/ml) for 24 h. (b, c) Real-time PCR (b) and western blot (c) were used to analyze the mRNA and protein levels of HSP70 after treatment with different concentrations of LPS (0, 200, 500, 1000, and 2000 ng/ml) for 24 h. ^∗^*P* < 0.05; ^∗∗^*P* < 0.01; ^∗∗∗^*P* < 0.001 vs. 0 ng/ml LPS. (d) ELISA was used to detect the secretory HSP70 after treatment with 500 ng/ml LPS for 0, 6, 12, and 24 h. (e, f) Real-time PCR (e) and western blot (f) were used to analyze the mRNA and protein levels of HSP70 after treatment with 500 ng/ml LPS for 0, 6, 12, and 24 h. ^∗^*P* < 0.05; ^∗∗^*P* < 0.01; ^∗∗∗^*P* < 0.001 vs. 0 h.

First, HUVEC were treated with different concentrations of LPS (0, 200, 500, 1000, and 2000 ng/ml) for 24 h. After incubation, the supernatant of treated cells was collected to perform ELISA and the cells were harvested to perform real-time PCR and western blot. The results showed that the secretory HSP70 was gradually decreased after treatment with different concentrations of LPS (Figure 1(a)). HSP70 was significantly downregulated after LPS stimulation at both the mRNA and protein levels (Figures 1(b) and 1(c)). Due to the changes in cell morphology after treatment with 1000 and 2000 ng/ml LPS, HUVEC were treated with 500 ng/ml LPS for 0, 6, 12, and 24 h subsequently. At each time point, the supernatant and treated cells were collected to measure the HSP70 level. As shown in Figure 1(d), 500 ng/ml LPS significantly decreased the secretory HSP70 at all time points, especially 24 h. Both the mRNA and protein levels of HSP70 were significantly downregulated after LPS stimulation at all time points. The highest inhibition rate was observed at 24 h (Figures 1(e) and 1(f)). Thus, 500 ng/ml LPS was used to treat cells for 24 h in the following experiments.

### 3.2. HSP70 Agonist Could Alleviate These Effects Caused by LPS

**Figure 2:**
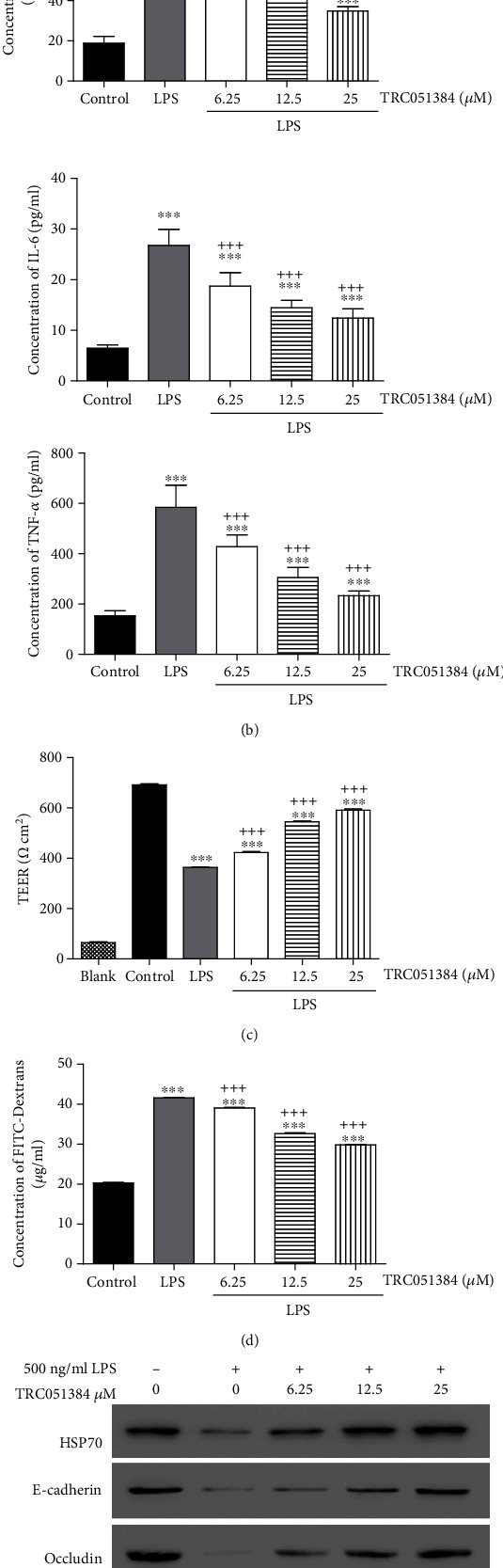
HSP70 agonist could alleviate the effects induced by LPS. (a) CCK-8 assay was used to measure cell viability in HUVEC under treatments with LPS and/or different concentrations of TRC051384. (b) ELISA was used to detect the productions of IL-1*β*, IL-6, and TNF-*α* after treatments. (c, d) Endothelial permeability was measured using TEER assay and FITC-Dextrans. (e) Expression levels of HSP70, E-cadherin, occludin, ZO-1, p38, and p-p38 were analyzed by western blot. ^∗∗∗^*P* < 0.001 vs. control; ^+^*P* < 0.05; ^+++^*P* < 0.001 vs. LPS.

To explore the role of HSP70 in HUVEC after LPS stimulation, the HSP70 agonist, TRC051384, was applied to treat cells. As shown in Figure 2(a), LPS markedly inhibited cell viability while TRC051384 could reverse this effect in a dose-dependent manner. Upon treatment with TRC051384, LPS-induced production of inflammatory cytokines including IL-1*β*, IL-6, and TNF-*α* was attenuated (Figure 2(b)).

Maintenance of normal cell permeability is an important function of vascular endothelial cells [9]. We next examined the changes of cell permeability after LPS stimulation using TEER assay and FITC-Dextrans. LPS significantly decreased the TEER value which could be attenuated by TRC051384 in HUVEC (Figure 2(c)). TRC051384 also alleviated LPS-induced increased level of FITC-Dextrans in HUVEC (Figure 2(d)). Thus, HSP70 can regulate LPS-induced augmentation of cell permeability of vascular endothelial cells *in vitro*. The cell junction is a key factor of maintenance of normal cell permeability in vascular endothelial cells [23]. To investigate the mechanism, we detected the expression of several cell junction proteins including E-cadherin, occludin, and ZO-1 using western blot. As shown in Figure 2(e), lower expression levels of E-cadherin, occludin, and ZO-1 were exhibited in the LPS-treated group, suggesting LPS stimulation inhibited the expressions of E-cadherin, occludin, and ZO-1 in HUVEC. Cotreatment with LPS and TRC051384 showed higher expressions of E-cadherin, occludin, and ZO-1 comparing with those in the LPS-treated group. These results indicated that the HSP70 agonist reversed the inhibition of cell junction proteins induced by LPS in HUVEC.

Recently, several reports indicate that p38 MAPK signaling is involved in the regulation of endothelial permeability [24, 25]. We found that LPS stimulation led to a markedly elevated phosphorylation level of p38, while TRC051384 could attenuate these effects (Figure 2(e)). Collectively, the HSP70 agonist could alleviate the effects induced by LPS, and p38 MAPK signaling may be involved in this process.

### 3.3. p38 Inhibitor Could Alleviate the Effects Induced by the HSP70 Inhibitor

**Figure 3:**
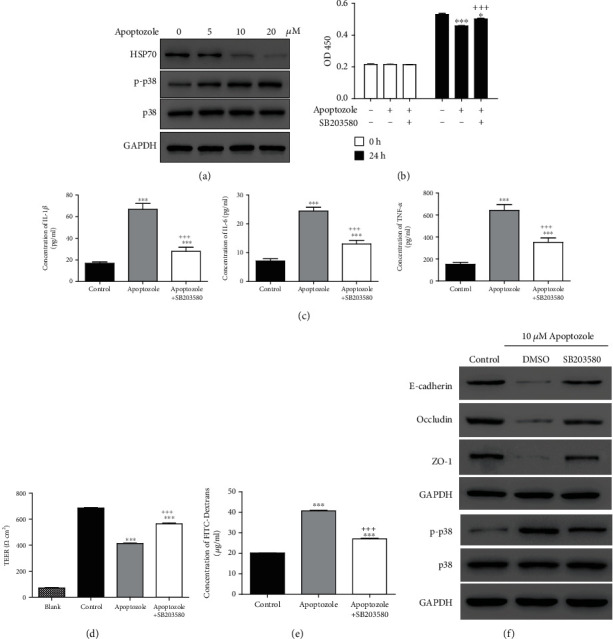
The effects induced by the HSP70 inhibitor could be blocked by the p38 inhibitor. (a) Expression levels of HSP70, p38, and p-p38 were analyzed in the cells treated with 0, 5, 10, and 20 *μ*M Apoptozole. (b) CCK-8 assay was used to measure cell viability in HUVEC under treatments with Apoptozole and/or SB203580. (c) ELISA was used to detect the productions of IL-1*β*, IL-6, and TNF-*α* after treatments. (d, e) Endothelial permeability was measured using TEER assay and FITC-Dextrans. (f) Expression levels of E-cadherin, occludin, ZO-1, p38, and p-p38 were measured by western blot. ^∗^*P* < 0.05; ^∗∗∗^*P* < 0.001 vs. control; ^+++^*P* < 0.001 vs. Apoptozole.

To certify our hypothesis whether p38 participated in the protection of HSP70 in sepsis, SB203580 (an inhibitor of p38) was used to treat HUVEC. First, the expression levels of HSP70, p-p38, and p38 were detected in the cells treated with Apoptozole, an inhibitor of HSP70. As shown in Figure 3(a), Apoptozole significantly downregulated HSP70 and upregulated p-p38 in a dose-dependent manner. Further, cells were treated with Apoptozole and/or SB203580, and then cell viability, productions of inflammatory cytokines, and cell permeability were analyzed. Interestingly, we found that Apoptozole markedly decreased cell viability but increased cell permeability and productions of IL-1*β*, IL-6, and TNF-*α*. However, these effects induced by Apoptozole could be attenuated by SB203580 (Figures 3(b)–3(d)). Subsequently, the lower levels of E-cadherin, occludin, and ZO-1 and the higher level of p-p38 were observed in the cells treated with Apoptozole comparing with the control group, while cells treated with SB203580 exhibited higher levels of E-cadherin, occludin, and ZO-1 and a lower level of p-p38 comparing with the Apoptozole group (Figure 3(f)). Taken together, HSP70 could regulate the phosphorylation level of p38, and these effects induced by the HSP70 inhibitor also could be alleviated by the p38 inhibitor.

### 3.4. HSP70 Increased Survival in the Mice with Sepsis

**Figure 4:**
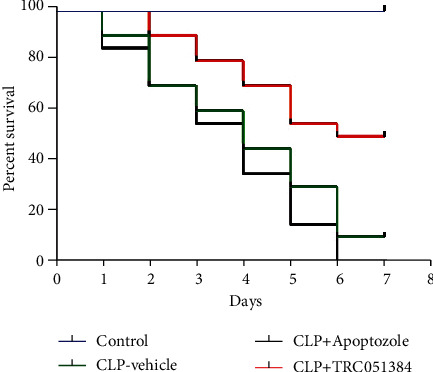
HSP70 increased the survival rate in mice with sepsis. Kaplan-Meier survival curves with Apoptozole or TRC051384 treatment after sepsis induction in mice. Mice were treated with the control, CLP, CLP+Apoptozole, (10 mg/kg i.p. 30 minutes before surgery), and CLP+TRC051384 (10 mg/kg i.p. 30 minutes before surgery) (*n* = 20).

CLP was used to establish the sepsis mouse model; then, mouse survival was recorded. As shown in Figure 4, septic mice treated with TRC051384 significantly increased survival compared with sepsis alone. However, septic mice treated with Apoptozole significantly decreased survival compared with sepsis. Our results suggested that HSP70 increased survival in the mice with sepsis.

## 4. Discussion

This study showed that LPS stimulation decreased the expression level of HSP70, which could reverse the damage effects induced by LPS in HUVEC. HSP70 was found to increase survival of sepsis in a mouse model established by CLP. Therefore, we concluded that HSP70 played a protective role in sepsis, indicating it maybe a novel therapy target for sepsis.

Sepsis is the first leading cause of death in noncoronary intensive care units, and it represents the systemic inflammatory response to infection [3]. Booming proinflammatory cytokines induced by severe infection damage the endothelium, which is considered the important hallmark of deterioration during sepsis [26]. Endothelial injury is associated with various critical illnesses and may lead to organ failure and mortality [27]. Injury of barrier function with increased cell permeability is considered microvascular dysfunction [28, 29]. In this study, LPS stimulation significantly increased the productions of inflammatory cytokines including IL-1*β*, IL-6, and TNF-*α* in HUVEC and highly inhibited cell viability (Figure 2). Then, we found that LPS markedly elevated cell permeability which was represented by the decrease of the TEER value and increase of FITC-Dextrans (Figure 2).

HSP70, the best known HSP, plays a protective role in sepsis-related lung injury [30]. It is reported that HSP70 promotes cell survival by inhibiting the death-associated permeabilization of lysosomes [30]. Furthermore, HSP70 inhibits cell apoptosis by decreasing permeabilization of mitochondrial membranes and thereby preventing Bax translocation [31]. In this study, HSP70 significantly decreased the level of FITC-Dextrans and increased the TEER value in HUVEC with LPS stimulation (Figure 2). Subsequently, we also observed that HSP70 reversed the inhibition of cell junction proteins, including E-cadherin, occludin, and ZO-1 induced by LPS (Figure 2). In addition, LPS-induced inhibition of cell viability and promotion of inflammatory cytokines can be blocked by HSP70 (Figure 2). Our results demonstrated that HSP70 showed its protective function in LPS-induced HUVEC by preventing cell permeability.

The mechanism of protection of vascular endothelial permeability by HSP70 remains unclear. As reported, HSP70 effectively downregulates the expressions of proinflammatory genes by interrupting the phosphorylation of transcription factors including p38 [18]. HSP70 is found to regulate p38 MAPK stability by interacting with MK2 [32]. In addition, p38 MAP kinase activities are involved in the regulation of thrombin-induced endothelial cell permeability [24]. Inhibition of p38 MAPK activity can attenuate LPS-induced hyperpermeability and pulmonary edema formation [25]. In this study, the increased level of p-p38 was observed with LPS stimulation and inhibition of p38. SB203580 could attenuate the effects induced by the HSP70 inhibitor (Figure 3). Our results indicated that p38 participated in the changes of cell permeability regulated by HSP70. Moreover, HSP70 increased survival of sepsis in a mouse model. Collectively, these data proved that HSP70 downregulated the level of p-p38 to reduce cell permeability, which had a crucial effect in protecting endothelial barrier function. In the current study, we revealed the function of HSP70 in the sepsis cell model, suggesting HSP70 maybe a therapy target for sepsis. However, we only explored the function of HSP70 in the sepsis cell model. To further estimate the role of HSP70, the sepsis mouse model should be involved in the following study. In addition, the deeper mechanism and whether some other signaling pathways are involved need to be further investigated.

## 5. Conclusions

Our results revealed that HSP70 played a protective role in sepsis by reducing the cell permeability and maintaining endothelial barrier function. HSP70 may be a potential novel therapy target of sepsis.

## Data Availability

The data used to support the findings of this study are available from the corresponding authors upon request.
